# Clinical significance of circulating tumor cell related markers in patients with epithelial ovarian cancer before and after adjuvant chemotherapy

**DOI:** 10.1038/s41598-021-88780-w

**Published:** 2021-05-18

**Authors:** Meysam Yousefi, Sara Rajaie, Vahideh Keyvani, Somayeh Bolandi, Malihe Hasanzadeh, Alireza Pasdar

**Affiliations:** 1 Department of Medical Genetics and Molecular Medicine, Faculty of Medicine, Mashhad University of Medical Sciences, Mashhad, Iran; 2 Medical Genetics Research Center, Mashhad University of Medical Sciences, Mashhad, Iran; 3 Department of Medical Genetics, Faculty of Medicine, Ahvaz Jundishapur University of Medical Sciences, Ahvaz, Iran; 4 Department of Biology, Islamic Azad University, Arsanjan Branch, Arsanjan, Iran; 5 Department of Biology, Faculty of Science, Shahid Chamran University of Ahvaz, Ahvaz, Iran; 6 Department of Gynecologic Oncology, Faculty of Medicine, Mashhad University of Medical Sciences, Mashhad, Iran; 7 Division of Applied Medicine, Faculty of Medicine, University of Aberdeen, Foresterhill, Aberdeen, UK

**Keywords:** Cancer, Molecular biology

## Abstract

Circulating tumor cells (CTCs) have recently been considered as new prognostic and diagnostic markers for various human cancers; however, their significance in epithelial ovarian cancer (EOC) remains to be elucidated. In this study, using quantitative real-time PCR, we evaluated the expression of *EPCAM*, *MUC1*, *CEA*, *HE4* and *CA125* mRNAs, as putative markers of CTCs, in the blood of 51 EOC patients before and/or after adjuvant chemotherapy. Our results demonstrated that, before chemotherapy, the expression of *EPCAM*, *MUC1*, *CEA* and *HE4* mRNAs were correlated to each other. *CEA* expression was correlated with tumor stage (r = 0.594, *p* = 0.000) before chemotherapy, whereas its expression after chemotherapy was correlated with serum levels of CA125 antigen (r = 0.658, *p* = 0.000). *HE4* mRNA showed the highest sensitivity both before and after chemotherapy (82.98% and 85.19%, respectively) and the persistence of this marker after chemotherapy was associated with advanced disease stage. The expression of *CA125* mRNA had negative correlation with the other markers and with tumor stage and therapy response (evaluated by the measurement of serum CA125 antigen). Collectively, our results indicated a better clinical significance of tumor-specific markers (*CEA* and *HE4* mRNAs) compared to epithelial-specific markers (*EPCAM* and *MUC1* mRNAs).

## Introduction

Ovarian cancer is the most lethal gynecologic malignancy and the fifth most frequent cause of cancer-related deaths worldwide^1^. According to the estimations from Surveillance, Epidemiology, and End Result (SEER) Program of the National Cancer Institute, about 22,000 new cases of ovarian cancer were diagnosed in the US in 2020, accounting for 1.2% of all new cancer cases. Furthermore, this daunting cancer makes about 14,000 people to succumb to their disease, which is estimated to be 2.3% of all cancer-related mortalities in the US in 2020^2^. Given the lack of specific symptoms and the paucity of satisfactory tests for the screening of ovarian cancer, about 70% of the patients are diagnosed at late stages when the tumor has metastasized to the peritoneal cavity and distant organs. Consequently, the 5-year overall survival (OS) rate of the patients is less than 50%. It has also been reported that when the tumor is still in an early stage, and is confined to the ovaries, less than 10% of the patients will die of the disease^2, 3^. Therefore, tumor dissemination and metastasis is the major cause of ovarian cancer-related deaths.

Circulating tumor cells (CTCs) are cancer cells that are shed from the tumors to the circulation. Although most of the CTCs are cleared by the immune system, a few of them may survive in the blood stream and disseminate to distant organs to form the metastatic disease^4^. Therefore, the detection of CTCs in the blood of patients with solid tumors may have valuable information with regard to an evident diagnostic and prognostic approach. For instance, studies have demonstrated that enumeration of CTCs in the blood could help stratify the cancer patients into high-risk and low-risk groups and serve as a prognostic factor for OS and progression-free survival (PFS) in breast, colorectal, prostate and lung cancers^5–8^. Recent studies have also revealed that characterization of CTCs could help predict therapy response^9–13^. Therefore, as a non-invasive approach which enables the possibility of longitudinal assessments, evaluation of CTCs in the blood would be a useful tool for evaluation of cancer progression and therapeutic efficiency.

In spite of a plethora of studies, which report the significance of CTCs in various human cancers, the prognostic significance of CTCs in ovarian cancer has not yet been robustly confirmed. It has been traditionally thought that ovarian cancer metastasis mostly occurs through the transcoelomic dissemination, i.e. the direct extension of the primary tumor to the peritoneal cavity and omentum by the ascitic fluid^3, 14, 15^. Consequently, the insight that the hematogenous metastasis is an uncommon metastatic route for ovarian cancer dissemination may have been a main reason for paucity of information about ovarian CTCs. However, given the recent updates that spread of ovarian tumor cells through the blood stream accounts for a large proportion of ovarian cancer metastases^15–17^, recent studies have reported that tracing of ovarian CTCs in the blood may be a promising approach^18^. According to recent studies, the detection of CTCs in the peripheral blood of ovarian cancer patients is correlated with the tumor stage, disease recurrence, shorter OS and PFS, the presence of ascites and sub-optimal debulking as well as the elevated levels of CA125 and HE4 proteins in serum^19–26^. Therefore, as the blood-borne dissemination of ovarian cancer from intraperitoneal tumor sites occurs earlier than the establishment of distant metastases, the detection of CTCs may herald a poor clinical outcome in ovarian cancer patients.

The most prominent approaches for the detection and/or isolation of CTCs include (1) immunological assays using monoclonal antibodies specific for epithelial markers, (2) PCR-based methods for the detection of tissue- or tumor-specific transcripts and (3) isolation by the size of the tumor cell (ISET)^27^. By using different methods for the detection of CTCs, studies have revealed the positivity rates of 12% to 90% for ovarian CTCs, highlighting the importance of the method and the markers selected for the detection of ovarian CTCs^19–26^. In the present study, using a quantitative real-time PCR (qPCR), we investigated the expression of a multi-marker gene panel, consisting of *EpCAM*, *MUC1*, *CEA*, *HE4* and *CA125* mRNAs as putative CTC markers in the blood of patients with ovarian cancer before the initiation of and after the completion of adjuvant chemotherapy. Our aim was to unravel whether ovarian CTC markers could help evaluate therapy response as well as their possible correlations with the clinico-pathological characteristics in ovarian cancer patients.

## Materials and methods

### Patient characteristics

The present study was conducted at the Ghaem Hospital, Mashhad University of Medical Sciences in Mashhad. In this study, 51 patients with histologically confirmed stages IA to IV epithelial ovarian cancer (EOC) were enrolled. The tumors were classified based on the World Health Organization (WHO) classification of tumors of the female genital tract, the tumor grading was classified according to Silverberg^28^ and the staging was conducted according to the Fédération Internationale de Gynécologie et d'Obstétrique (FIGO)^29^. Additionally, 14 age-matched healthy women were included to the study as controls. Informed written consent was obtained from all the patients and the healthy volunteers. All the methods and protocols were performed in accordance with the guidelines and regulations of Mashhad University of Medical Sciences. The study was approved by the Ethics Committee of Mashhad University of Medical Sciences (ethical code IR.MUMS.MEDICAL.REC.1397.462).

The mean and median ages of the patients were 54.02 and 53 years, respectively (range, 34–81 years). The clinical diagnosis of the patients was confirmed by histopathology and the histological subtypes were serous (n = 43), mucinous (n = 5), or other subtypes (n = 3). The whole patients’ population was subjected to primary radical surgery, including abdominal hysterectomy, bilateral salpingo-oophorectomy, peritoneal stripping, infragastric omentectomy, and pelvic as well as paraaortic lymphadenectomy, where possible. The most important aim of the surgery was to macroscopically resect the tumor content as much as feasible. All patients received adjuvant chemotherapy (combination of Carboplatin and Paclitaxel; two received Carboplatin alone) and the tumors were defined as platinum-resistant when they recurred less than 6 months after the completion of the platinum-based chemotherapy. Patients’ characteristics are shown in Table 1.

**Table 1:** Clinicopathological characteristics of the EOC patients.

Characteristic	No. (%)
Total No. of patients	51 (100)
**Age (year)**	
Mean	54.02
Median	53
Range	34–81
**FIGO stage**	
I	10 (19.6)
II	9 (17.7)
III	30 (58.8)
IV	2 (3.9)
**Grading**	
I	3 (5.9)
II	10 (19.6%)
III	38 (74.5)
**Histologic subtype**	
Serous	43 (84.3)
Mucinous	5 (9.8)
Other	3 (5.9)
**Serum CA125 level**	
< 35 U/ml	2 (3.9)
≥ 35, < 100 U/ml	7 (13.7)
≥ 100 U/ml	42 (82.4)
**Chemotherapy**	
Platinum-based	51 (100)
**Debulking status**	
Optimal (≤ 1 cm)	32 (62.7)
Suboptimal (≥ 1 cm)	19 (37.3)

### Blood sampling and PBMC isolation

The peripheral blood specimens were taken from 48 patients at the beginning of adjuvant chemotherapy and from 27 patients 2–3 weeks after the completion of 6 cycles of chemotherapy, as well as from the healthy controls. For each individual, 6 ml of peripheral blood was collected in a blood collection tube containing EDTA. In order to avoid the contamination of the blood with the surrounding epidermal (epithelial) cells, the blood specimens were obtained from the middle of the vein punctures after discarding the first 2–3 ml of blood. For peripheral blood mononuclear cells (PBMCs) isolation, the whole blood sample from each participant was diluted 1:2 times in phosphate buffered saline (PBS) and then subjected to density gradient (1.077 g/mol) centrifugation by 3 ml Ficoll-Hypaque (Pharmacia, Freiburg, Germany) at 800 g for 20 min. The interface between serum and Ficoll was transferred to a fresh tube and washed 3 times with PBS at 500 g for 10 min.

### RNA extraction, cDNA synthesis and qPCR

We used five CTC related molecular markers, *EPCAM*, *MUC1*, *CEA*, *HE4* and *CA125* mRNAs, for the detection of CTCs in the blood of EOC patients before and after adjuvant chemotherapy.

Total RNA was extracted from PBMCs using RNX-Plus solution (SinaClone BioScience, Tehran, Iran) according to the manufacturer’s instructions. The quality of the RNA samples was checked by electrophoresis on 2% agarose gel, followed by quantification via NanoDrop ND-1000 (Nanodrop Technologies, Wilmington, Delaware, USA). From each RNA sample, 0.5–2 µg was reverse-transcribed into cDNA using cDNA synthesis kit (Yekta Tajhiz Azma, Tehran, Iran) according to the manufacturer’s instructions. Thereafter, qPCR was performed with a LightCycler 96 instrument (Roche Diagnostics GmbH, Germany) using 10 µl 2X SYBR green master mix (Biofact, Daejeon, South Korea), plus 2 μl of cDNA samples, 0.5 μl of each forward and reverse primers (10 pmol) and 7 μl of nuclease-free water. The thermal cycling conditions involved an initial step of 15 min at 95 °C for enzyme activation, followed by 40 cycles including a denaturation step for 30 s at 95 °C, an annealing step for 30 s at 58 °C and an extension step for 30 s at 72 °C. In the PCR protocol, pooled cDNA from OVCAR-3, CAOV-3 and A2780 cell lines were used as positive control; and nuclease-free water was used as the negative control. To assess the efficiency and sensitivity of the qPCR assays, standard curves were plotted for each marker’s mRNA. For this purpose, briefly, the pooled cDNA sample was used as template to amplify the intended mRNAs by PCR, followed by purification of the PCR products by extracting the desired bands from agarose gel and determining the copy numbers per ml using Avogadro’s law. Serial dilutions (10^2^ to 10^6^ copies per µl) were then prepared and used as templates in each single run of qPCR. Melting curve analysis was applied to verify the single PCR product of each primer set. In this study, hypoxanthine phosphoribosyltransferase1 (*HPRT1*) was amplified as the reference gene and the relative expression for each target mRNA was calculated according to the relative expression method (2^−ΔΔCq^). The sequences and other details of the primers are given in Table 2.

**Table 2:** The nucleotide sequences of primers used for the qPCR assay.

Gene	Accession number	Forward primer (5ʹ–3ʹ)	Reverse primer (5ʹ–3ʹ)	Amplicon size
HPRT1	NM_000194	CCTGGCGTCGTGATTAGTGAT	AGACGTTCAGTCCTGTCCATAA	131
EPCAM	NM_002354	ATAACCTGCTCTGAGCGAGTG	TGCAGTCCGCAAACTTTTACTA	99
MUC1	NM_002456.4	TCGTAGCCCCTATGAGAAGG	CCACTGCTGGGTTTGTGTAA	71
CEA	NM_001291484	GGCCACTGTCGGCATCAT	GGAAGAAGCAAAACAACTGTCAGTC	108
HE4	NM_006103	CGGCTTCACCCTAGTCTCAG	CCTCCTTATCATTGGGCAGA	173
CA125	NM_024690	GCTACCACAGGTTCCAGTCC	CGACGGTTATAACCTGTAGCG	248

### Statistical analyses

To determine the levels of the markers which best differentiate EOC cases versus the controls, receiver operating characteristic (ROC) curves, area under the curve (AUC), sensitivity, specificity, and the likelihood ratios (LRs) were calculated. The levels of markers that maximized the sensitivity and specificity were considered as the optimal cut-off values. The LR was calculated according to the formula sensitivity/1- specificity. The differences between the groups were assessed by the non-parametric Mann–Whitney U test. The Spearman exact test was performed to evaluate the correlation between the expression of the markers either before or after adjuvant chemotherapy and the histopathological characteristics of the patients. The differences between the rates of the groups were compared by Fisher’s exact test or Pearson’s χ^2^ test. *p* Value of < 0.05 was defined as statistically significant. Statistical analyses were performed via GraphPad Prism 8.0 (GraphPad Inc. San Diego, CA, USA) and IBM SPSS Statistics 25 (SPSS, Chicago, IL, USA).

## Results

**Figure 1:**
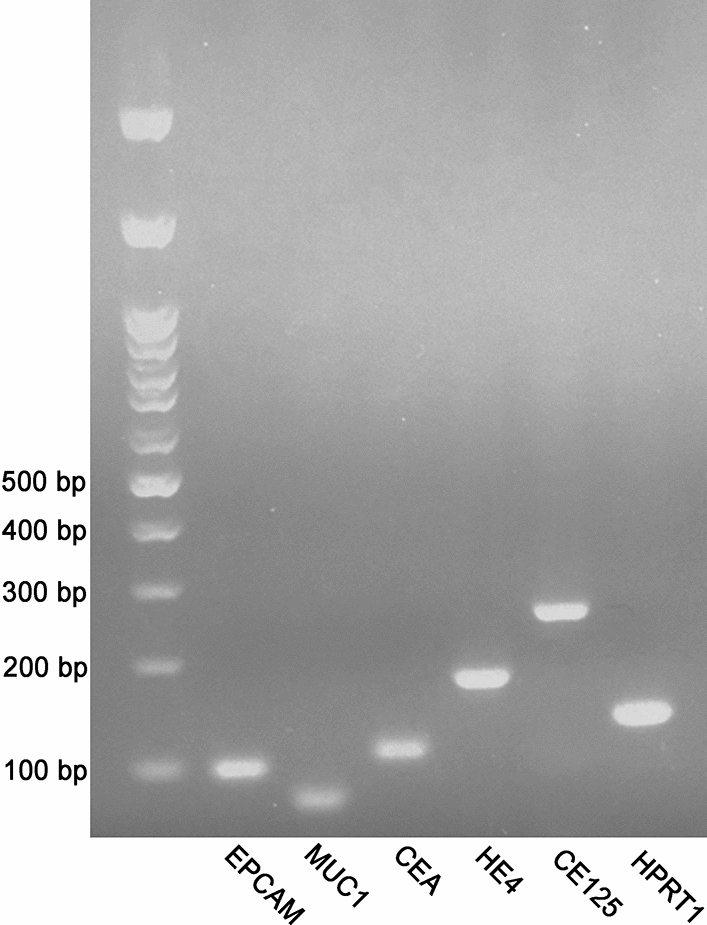
Gel electrophoresis image (agarose 2%) of the amplification products obtained for our target genes. From the left, lane 1: molecular weight marker (100-pb DNA ladder); lanes 2–7: *EPCAM* (99 bp), *MUC1* (71 bp), *CEA* (108 bp), *HE4* (173 bp), *CA125* (248 bp) and *HPRT1* (131 bp).

**Figure 2:**
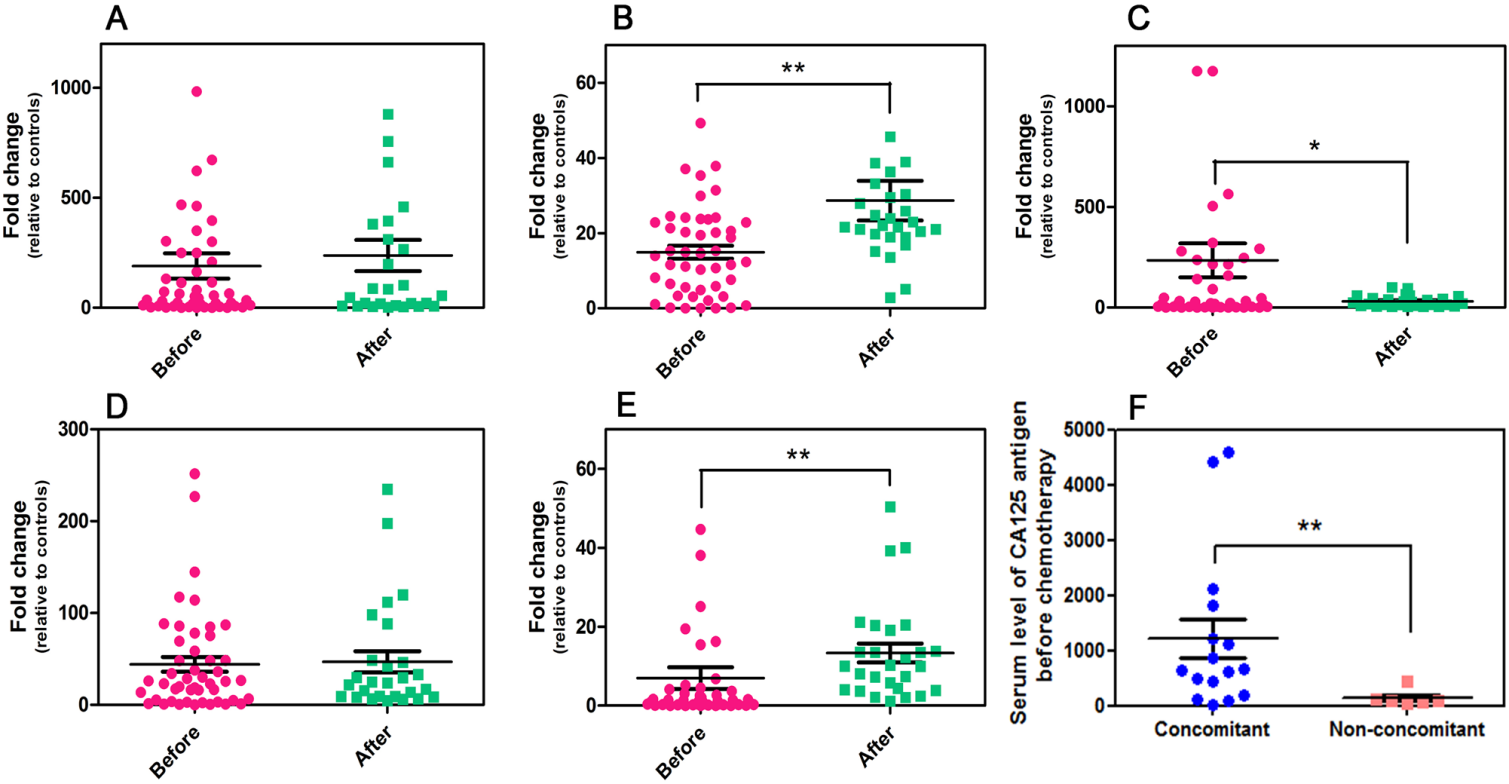
Relative expression levels of (**A**) *EPCAM*, (**B**) *MUC1*, (**C**) *CEA*, (**D**) *HE4* and (**E**) *CA125* mRNAs in EOC patients before and after adjuvant chemotherapy. (**F**) Concomitant detection of *HE4* mRNA before and after chemotherapy is associated with higher levels of serum CA125 antigen (measured before chemotherapy) (*: *p* < 0.05, **: *p* < 0.01).

In this study, we amplified the *EPCAM*, *MUC1*, *CEA*, *HE4*, *CA125* and *HPRT1* mRNAs using qPCR and the correct band size of each target gene was confirmed by gel electrophoresis (Fig. 1). The amplification and efficiency of qPCR assay were also confirmed by analyzing the melting curve and the standard curve slopes. The standard curves demonstrated that our qPCR assays were sensitive enough to amplify even a few numbers of DNA copies (Supplementary Fig. 1). The relative quantification of mRNA expressions using qPCR demonstrated that the expressions of all five markers, either before or after chemotherapy, were significantly higher compared to their respective controls (*p* ˂ 0.05). Comparing the average expression of each marker before and after chemotherapy revealed that the expression of *CEA* was significantly decreased after the administration of chemotherapy (*p* ˂0.05). However, chemotherapy did not alter the expression of *EPCAM* and *HE4*, while, unexpectedly, it led to increased expression of *MUC1* and *CA125* (*p* ˂ 0.01) (Fig. 2A–E).

### The sensitivity, specificity and area under the curves of the markers in EOC patients before and after adjuvant chemotherapy

**Figure 3:**
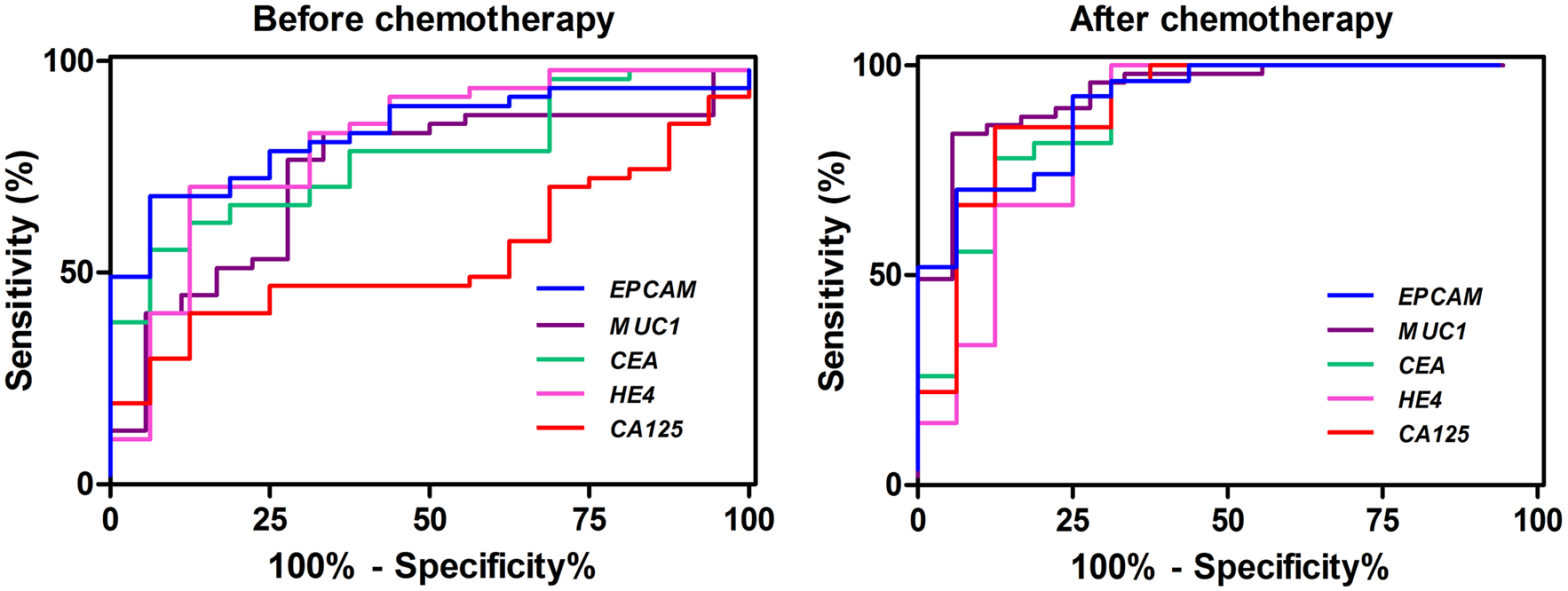
Receiver operating characteristic (ROC) curves for *EPCAM*, *MUC1*, *CEA*, *HE4* and *CA125* mRNA markers before and after chemotherapy.

In an attempt to assess the ability of molecular discrimination between the controls and the patients either before or after chemotherapy, the ROC curve analysis was carried out for each marker. The optimal cut-off for each biomarker was determined according to the best balance between the sensitivity and specificity and the larger LR. Table 3 shows the cut-off point, sensitivity, specificity and AUC of each biomarker for the patients before and after adjuvant chemotherapy. Also, the ROC curves for the five markers before and after chemotherapy are given in Fig. 3. The AUC analysis provided high levels of sensitivity and specificity for all of the markers except *CA125* mRNA. Before the initiation of chemotherapy, the highest sensitivity was obtained for *HE4* (82.98%), followed by *EPCAM* (80.85%), *MUC1* (80.98%) and *CEA* (78.72%). Specificity level for these cut-off points were 68.75% for *HE4* and *EPCAM* and 62.50% for *MUC1* and *CEA*, whereas the LR indicates that the levels greater than or equal to the cut-off points were at least 2.21 times as likely to be found in the patients than within the controls. The lowest sensitivity before chemotherapy was obtained for *CA125* (29.79%) although it had the highest specificity compared to other markers (93.75%; cut-off 0.301, AUC 0.540). After chemotherapy, again the highest sensitivity was obtained for *HE4* (85.19%) at a cut-off point where the specificity was 75.00% (Table 3). The combinations of biomarkers were also evaluated using a multiple logistic regression model. These combinations did not improve the AUC from the model by each marker alone (data not shown).

**Table 3:** The sensitivity, specificity, LR and AUC of ROC of *EPCAM*, *MUC1*, *CEA*, *HE4* and *CA125* markers in EOC patients before and after adjuvant chemotherapy.

Before chemotherapy						After chemotherapy					
mRNA marker	Cut-off point	Sensitivity (%)	Specificity (%)	LR	AUC	mRNA marker	Cut-off point	Sensitivity (%)	Specificity (%)	LR +	AUC
*EPCAM*	>4.355	80.85	68.75	2.59	.820	*EPCAM*	>16.220	70.37	93.75	11.26	.907
*MUC1*	>2.928	82.98	62.50	2.21	.735	*MUC1*	>13.39	82.59	93.75	14.81	.938
*CEA*	>3.248	78.72	62.50	5.51	.773	*CEA*	>8.715	77.78	87.50	6.74	.887
*HE4*	>2.730	82.98	68.75	2.66	.806	*HE4*	>7.708	85.19	75.00	3.41	.854
*CA125*	>0.301	29.79	93.75	4.77	.540	*CA125*	>3.605	85.19	87.50	6.81	.901

### The correlation between the expression of *EPCAM*, *MUC1*, *CEA*, *HE4* and *CA125* mRNAs before and after adjuvant chemotherapy

We studied the correlation between the mRNA levels obtained for all the five markers before and after adjuvant chemotherapy. As shown in Table 4, before chemotherapy, the expression of *EPCAM*, *MUC1*, *CEA* and *HE4* were significantly correlated to each other, as examined by the Spearman rank test. The highest correlation was obtained for *CEA* and *HE4* (r = 0.694, *p* = 0.000), followed by *CEA* and *EPCAM* (r = 0.643, *p* = 0.000). However, the expression of *CA125* was negatively correlated with the other four markers, although its negative correlation was statistically significant only for *MUC1* expression (r =  − 0.37, *p* = 0.01). After chemotherapy, the expression of *CEA* had a correlation with the expression of *EPCAM* (r = 0.46, *p* < 0.05) and *HE4* (r = 0.44, *p* < 0.05) (Table 4). In addition, *CA125* expression showed a similar behavior after chemotherapy with that of before chemotherapy in terms of correlation with other markers, as it was almost unrelated to the expression of the other markers. The expression of neither of the markers before chemotherapy was correlated to that marker itself or the other markers after chemotherapy (data not shown).

**Table 4:** The Spearman rank correlations between mRNA expressions of *EPCAM*, *MUC1*, *CEA*, *HE4* and *CA125* markers in EOC patients before and after adjuvant chemotherapy.

Before chemotherapy						After chemotherapy					
mRNA marker	*EPCAM*	*MUC1*	*CEA*	*HE4*	*CA125*	mRNA marker	*EPCAM*	*MUC1*	*CEA*	*HE4*	*CA125*
*EPCAM*	1.000 –	.349 (.016)	.643 (.000)	.538 (.000)	− .259 (.079)	*EPCAM*	1.000 –	− .280 (.158)	.461 (.015)	− .092 (.650)	− .024 (.907)
*MUC1*	.349 (.016)	1.000 –	.534 (.000)	.482 (.001)	− .369 (.011)	*MUC1*	− .280 (.158)	1.000 –	.152 (.450)	.105 (.603)	.236 (.236)
*CEA*	.643 (.000)	.534 (.000)	1.000 –	.694 (.000)	− .155 (.300)	*CEA*	.461 (.015)	.152 (.450)	1.000 –	.443 (.021)	− .065 (.747)
*HE4*	.538 (.000)	.482 (.001)	.694 (.000)	1.000 –	− .213 (.151)	*HE4*	− .092 (.650)	.105 (.603)	.443 (.021)	1.000 –	.059 (.770)
*CA125*	− .259 (.079)	− .369 (.011)	− .155 (.300)	− .213 (.151)	1.000 –	*CA125*	− .024 (.907)	.236 (.236)	− .065 (.747)	.059 (.770)	1.000 –

*p* values are in parentheses.

### The correlation between the expression of the markers and clinicopathological characteristics of the EOC patients

The relations between the expression of the mRNA markers and clinicopathological features of the patients, including age, tumor grade, FIGO stage, serum CA125 protein level, histologic subtype and debulking status, were evaluated. Before chemotherapy, there were statistically significant correlations between the FIGO stage and the expression of *EPCAM* (r = 0.347, *p* = 0.017), *MUC1* (r = 0.374, *p* = 0.010), and *HE4* (r = 0.335, *p* = 0.022), and especially *CEA* (r = 0.594, *p* = 0.000). However, the correlation between the expression of *CA125* and FIGO stage was not significant (r =  − 0.060, *p* = 0.690). The expression of *CA125* mRNA also had a negative correlation with serum levels of CA125 protein (r =  − 0.306, *p* = 0.036), further highlighting the different manner of *CA125* mRNA expression, compared to the other markers.

After chemotherapy, the expression of *CEA* mRNA showed a correlation with the serum level of CA125 protein (r = 0.658, *p* = 0.000), whereas *CA125* mRNA expression, similar to that of before chemotherapy, had a negative correlation with serum levels of CA125 antigen (r =  − 0.382, *p* = 0.049) and FIGO stage (r =  − 0.432, *p* = 0.024). The other correlations were not statistically significant (Supplementary Table 1).

### Concomitant detection of each marker before and after adjuvant chemotherapy

According to the detection status of the markers before and after chemotherapy, for each marker, patients were categorized into four subgroups: persistently positive patients, persistently negative patients, patients with positive pre-chemotherapy status changing to negative, and patients with negative pre-chemotherapy status changing to positive. The Pearson’s χ^2^ test revealed that the proportion of the patients with advanced FIGO stages (III + IV) in the persisting *HE4* mRNA-positive subgroup was significantly higher than in the other *HE4* subgroups (*p* = 0.0096). In addition, in persisting *HE4* mRNA-positive group, the average level of serum CA125 protein (measured before chemotherapy) was significantly higher than in the other *HE4* subgroups (1227.1 U/ml vs. 142.9 U/ml, *p* = 0.0045) (Fig. 2F).

## Discussion

Ovarian cancer is the deadliest gynecologic cancer and causes a great deal of burdens upon healthcare systems^1^. Up to 90% of ovarian cancers are arisen from the neoplastic transformation of ovarian surface epithelium and are usually referred to as EOC. This type of ovarian cancer accounts for the major cause of gynecologic cancers-related mortalities^30^. Despite improvements in the response rate of EOC patients with surgery followed by platinum-based chemotherapy, ultimately, more than 50% of the patients die of the complications associated with the disease progression. This survival rate drops to less than 20% once the disease is diagnosed in stages III and IV^31^. Therefore, the need for finding reliable biomarkers for early detection and monitoring of EOC patients, which are both sensitive and specific, remains a long-awaited priority. EOC management could be well supported by the application of biomarkers for early diagnosis, discriminating malignant tumors from benign pelvic masses, estimating prognosis, monitoring the treatment and predicting response to individual drugs^32^. In an attempt to explore the significance of CTC markers in ovarian cancer, we investigated the expression of *EPCAM*, *MUC1*, *CEA*, *HE4* and *CA125* mRNAs. Our results demonstrated a relatively high sensitivity for *EPCAM*, *MUC1*, *CEA* and *HE4* before and after chemotherapy, and significant correlations of these markers with each other along with correlation of *CEA* and *HE4* with patients’ characteristics.

Cancer antigen 125 (CA125) and human epididymis protein 4 (HE4) are the most widely used tumor markers in EOC. Serum levels of CA125 is often considered as the gold standard for the detection and monitoring of patients during therapy^33^. This high-molecular weight glycoprotein, which is expressed by coelomic and Mullerian epithelia, is raised in approximately 90% of patients with advanced EOC^34^. However, elevation of CA125 antigen is also observed in other physiological or pathological conditions, including pregnancy, menstruation, endometriosis and inflammatory diseases of the peritoneum^35^. In addition, studies have indicated the inadequate sensitivity of CA125 antigen for the detection of asymptomatic EOC. The elevation of CA125 antigen is observed in only 50% to 64% of stage I EOC patients^36, 37^, whereas CTCs can be detected in more than 90% of EOC patients with stage IA-IB disease^38^. HE4 is a relatively new biomarker which has recently been considered for diagnosing ovarian malignancies^39^. This glycoprotein belongs to the family of whey acidic four-disulfide core proteins (WAP), which is upregulated in ovarian tumors, especially in endometrioid ovarian cancer^40^. Studies have reported the specificity of 86% to 94% for HE4; which is much higher than CA125 (53% to 84%), mainly because its level is not affected by endometriosis^41^. However, low sensitivity of this marker has led to the development of algorithms, RMI (Risk of Malignancy Index) and ROMA (Risk of Ovarian Malignancy Algorithm) to improve the inherent characteristics of CA125 and HE4 markers. Given the significance of CA125 and HE4 for the diagnosis of patients with ovarian cancer, we evaluated the expression of their mRNAs as CTC markers in the blood of EOC patients. Our results demonstrated the higher sensitivity for *HE4* mRNA, in comparison with other CTCs investigated, both before and after chemotherapy (82.98 and 85.19% respectively). Guo et al. identified and counted CTCs in ovarian cancer patients using microfluidic isolation and immunofluorescent staining of CD45, HE4, and epithelial and mesenchymal markers^42^. Their results demonstrated a sensitivity of 73.3% for these HE4 + CTCs. However, they reported the lower specificity for HE4 + CTCs compared to CA125 antigen; therefore, they considered HE4 + CTCs and CA125 combined in screening ovarian cancer patients. Consequently, the specificity was relatively high (86.7%) in patients with elevated CA125 level. In line with this study, we observed specificity of 68.75% and 75.00% for *HE4* mRNA before and after chemotherapy, respectively. The persistence of HE4 mRNA detection after chemotherapy was associated with advanced stage of the disease and elevated CA125 antigen (measured before chemotherapy). Taken together, our results showed a reasonable sensitivity for *HE4* mRNA and the important correlations with the clinicopathological characteristics of EOC patients. However, as described by Guo et al., low specificity can be improved by combination of *HE4* mRNA with other biomarkers which are applied for EOC diagnosis. However, the expression of *CA125* mRNA lacked the sensitivity and also negatively correlated with *HE4* mRNA and the other three mRNAs and with CA125 antigen before and after chemotherapy. The lack of a direct correlation between the levels of *CA125* mRNA in blood and its protein in serum may be due to their different entities which necessitate the use of different methods to detect them^43^. In addition, CA125 protein is secreted by the cancerous cells in the primary tumor, whereas the *CA125* mRNAs are not directly released from the primary tumor into the circulation; they are rather extracted from the CTCs which are already shed from the primary tumor into the circulation and present in minute quantities in the blood.

Epithelial markers are expressed in carcinomas because these tumors originate from epithelial tissues. However, these markers are usually not expressed in normal blood cells since these cells have a mesenchymal origin^44, 45^. Therefore, the enrichment and detection of CTCs according to the detection of epithelial markers has vastly been used in the last decade. For instance, CellSearch platform, which is the only US Food and Drug Administration (FDA)-approved system for the detection of CTCs, takes advantage of positive immunostaining for epithelial cell adhesion molecule (EpCAM) and negative immunostaining for a common leukocyte antigen, CD45, to exclude leukocytes. Mucin 1 (MUC1) is another well-known epithelial marker for the detection of CTCs. This transmembrane glycoprotein is involved in the formation of mucin networks found in the secreted mucus gels^46^. The extracellular domain of MUC1 can serve as a ligand for endothelial cell receptors and, consequently, overexpression of this protein in cancer cells is usually associated with increased migration and invasion and commonly used as a biomarker for detection of carcinoma cells^47, 48^. In our study, expression of *EPCAM* and *MUC1* mRNAs had correlations with each other and with *CEA* and *HE4* mRNAs and FIGO stage before chemotherapy (Table 4); however, their expression was not correlated with therapeutic response, evaluated by comparison with serum levels of CA125 antigen.

Carcinoembryonic antigen (CEA), which is encoded by *CEA* gene (also known as cluster of differentiation (CD) 66e or *CEACAM5*), is a glycoprotein which has been shown to be expressed in embryonic tissues as well as a vast majority of human cancers, including colorectal, gastric, pancreatic, ovarian, breast and lung cancers, but is absent in normal adult tissues^49^. The glycoprotein product of *CEA* consists of a structure which is similar to that of the immunoglobulin superfamily and is thought to be involved in adhesion to the extracellular matrix and to other cell types thanks to the homophilic and heterophilic interactions with CD66a (CEACAM1) and CD66c (CEACAM6)^50^. In patients with colorectal cancer, the expression of CEA is associated with the stage of the disease^51^, so that elevated levels of CEA (exceeding 20 ng/ml) is usually associated with distant metastasis^52^. In case of EOC patients, elevated levels of serum CEA is reported to be observed in approximately 35% of the patients and occurs more often in mucinous subtypes (88%) than in serous subtypes (19%)^53, 54^. Monitoring of CEA in response to chemotherapy has been reported to be an indicator of therapeutic response and recurrence assessments^55^. Several studies have confirmed *CEA* mRNA as a reliable marker for the detection of CTCs in gastrointestinal cancers^56, 57^. For instance, using qPCR, it has been demonstrated that the expression of *CEA* mRNA in the peripheral blood of the patients with gastrointestinal and breast cancers before surgery is less than 20%^56^. However, after surgery its expression is detected in at least 46% of patients with gastrointestinal cancer^57^, which is because surgical procedure itself can cause the release of CTCs into the blood stream^58^. Given the significance of CEA in the assessment of response to therapy and recurrence, and that the present study consisted of CTC marker evaluations before and after adjuvant chemotherapy, we were interested to evaluate the dynamics of *CEA* mRNA expression. We observed that *CEA* mRNA was detected in 78.72% of the patients before chemotherapy and 77.78% of the patients after chemotherapy. One reason for this higher sensitivity rate in comparison to the mentioned studies may be different entity of the tumors evaluated, as there is a big heterogeneity observed in different human cancers and even in different subtypes of one cancer type in terms of CTC characteristics. Interestingly, among the five CTC markers investigated in this study, *CEA* mRNA was the only marker which expression was decreased after chemotherapy in comparison to that of before chemotherapy (*p* < 0.05). The expression of *CEA* mRNA before chemotherapy was correlated with tumor stage (r = 0.594, *p* = 0.000), while its expression after chemotherapy was correlated with CA125 antigen (r = 0.658, *p* = 0.000), the most important indicator of therapy response in EOC patients. These data indicate that *CEA* mRNA may be a reliable marker for the detection of CTCs in EOC patients and may have significance in terms of prognostication and evaluation of therapy response in these patients.

In conclusion, this is the first study evaluating a multi-marker panel of mRNAs, including tumor cell-specific (*CEA*, *CA125* and *HE4*) and epithelial-specific (*EPCAM* and *MUC1*) mRNAs in EOC patients before and after adjuvant chemotherapy. Unlike *CA125* mRNA, which had a totally different expression manner, the other markers’ expressions, were correlated to each other before chemotherapy and had reasonable sensitivity and specificity, which may shed lights into unraveling CTC characteristics in EOC patients. The combinations of the markers did not improve the AUC from the model by each marker alone. However, aggregating the results of these CTC markers with other biomarkers which are currently used for diagnosis and monitoring of EOC patients may help find new combinations of markers with improved sensitivity and specificity. *CEA* and *HE4* mRNAs showed a relatively greater value, because: (1) their expression were significantly correlated to each other both before and after chemotherapy, (2) the expression of *CEA* mRNA before chemotherapy had the most significant correlation with tumor stage (r = 0.594, *p* = 0.000), while, after chemotherapy, its expression was significantly decreased and showed correlation with CA125 antigen, and (3) *HE4* mRNA showed the highest sensitivity both before and after chemotherapy and the persistence of this marker was associated with advanced stages of the disease. One reason for the stronger observations obtained for tumor-specific markers (*CEA* and *HE4* mRNAs) may be the fact that EOC CTCs are heterogeneous and epithelial markers (i.e. *EPCAM* and *MUC1* mRNAs) may not represent all parts of the tumor. In addition, epithelial markers may be influenced by epithelial-mesenchymal transition and, therefore, their expressions may be diminished in a major part of CTCs. However, further studies and larger cohort sizes are demanded to confirm the clinical relevance of CTC mRNA markers in EOC patients.

## Supplementary Information

Supplementary Information.

## Data Availability

All data and materials are available upon request from the corresponding author.
