# Supplementary material for: Clinical significance of circulating tumor cell related markers in patients with epithelial ovarian cancer before and after adjuvant chemotherapy
Source: Sci Rep. 2021 May 18;11:10524. doi: 10.1038/s41598-021-88780-w (PMC8131620; doi:10.1038/s41598-021-88780-w)
Supplement: Supplementary file 1 — Supplementary Information. [file 41598_2021_88780_MOESM1_ESM.docx]

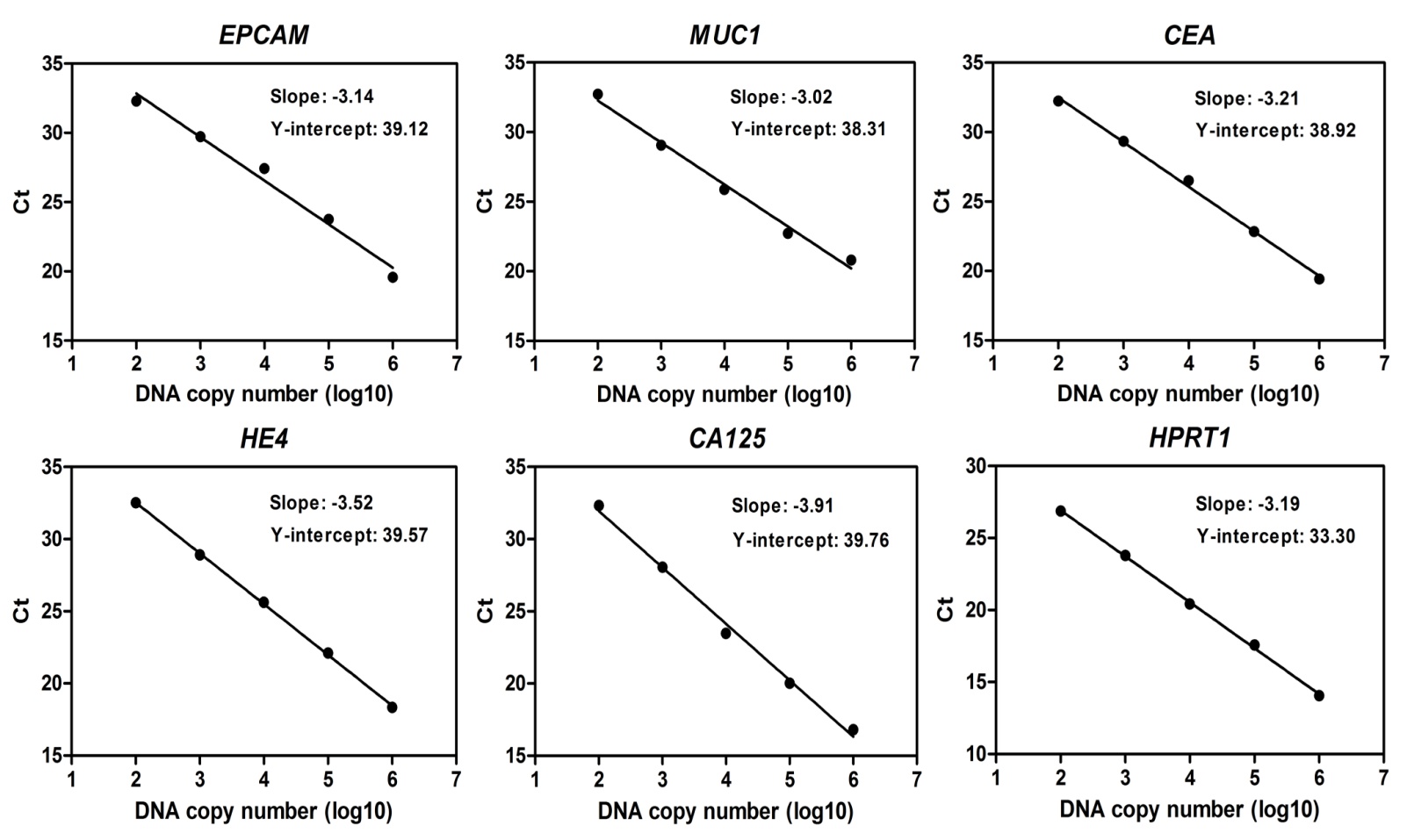


**Supplementary Fig. 1** Standard curves for amplification of the CTC-related cDNA markers using quantitative real-time PCR

**Supplementary Table 1** The correlations between the expressions of CTC markers and the clinic-pathological characteristics of EOC patients

|  | **Marker** | **Age** | **FIGO stage** | **Tumor grade** | **Histologic subtype** | **CA125 antigen**  **in serum**  **before chemotherapy** | **CA125 antigen**  **in serum**  **after chemotherapy** |
| --- | --- | --- | --- | --- | --- | --- | --- |
| **Before chemotherapy** | ***EPCAM*** | −.193  (.377) | .347  (.017) | .210  (.124) | .051  (.757) | .040  (.858) | .105  (.635) |
|  | ***MUC1*** | .116  (.599) | .374  (.010) | .046  (.340) | .097  (.082) | .219  (.316) | −.181  (.408) |
|  | ***CEA*** | −.140  (948) | .594  (.000) | .171  (.810) | .164  (.773) | .192  (.381) | .075  (.734) |
|  | ***HE4*** | .164  (.455) | .335  (.022) | .207  (.087) | .227  (.060) | .313  (.146) | −.133  (.547) |
|  | ***CA125*** | .006  (.977) | −.060  (0.690) | .078  (.201) | .010  (.441) | −.306  (.036) | −.222  .309 |
| **After chemotherapy** | ***EPCAM*** | .138  (.530) | −.140  (.523) | .063  (.922) | .210  (.118) | −.126  (.565) | .292  .176 |
|  | ***MUC1*** | −.011  (.961) | .030  (.892) | .097  (.330) | .218  (.281) | .213  (.330) | .215  (.326) |
|  | ***CEA*** | −.113  (.607) | .161  (.464) | .220  (.131) | .175  (.770) | .045  (.837) | .658  (.000) |
|  | ***HE4*** | −.225  (.053) | .352  (.100) | −.092  (.454) | .109  (.280) | .86  (.697) | .285  (.188) |
|  | ***CA125*** | .231  (.289) | −.432  (.024) | .170  (.342) | .010  (0.733) | -.102  (.644) | −.382  (.049) |

P values are in parentheses
